# MRI Characteristics of the Evolution of Supratentorial Recent Small Subcortical Infarcts

**DOI:** 10.3389/fneur.2015.00118

**Published:** 2015-05-26

**Authors:** Shuhei Okazaki, Eva Hornberger, Martin Griebe, Achim Gass, Michael G. Hennerici, Kristina Szabo

**Affiliations:** ^1^Department of Neurology, UniversitätsMedizin Mannheim, University of Heidelberg, Mannheim, Germany

**Keywords:** small subcortical infarcts, white matter hyperintensities, lacunes, cavity formation, infarct volume reduction, MRI

## Abstract

**Objective:**

Morphological changes of recent small subcortical infarcts are not well defined. The purpose of the present study was to describe the MRI characteristics of the evolution for this stroke subtype.

**Methods:**

We conducted a retrospective review of patients diagnosed with definite supratentorial recent small subcortical infarcts according to the ASCO classification with baseline and follow-up MRI (≥90 days of stroke onset). We investigated the incidence of cavity formation, the infarct volume change, and the positional relationship between infarct lesions and preexisting white matter hyperintensities (WMHs) of presumed vascular origin.

**Results:**

We identified 62 patients with a median age of 71 years (range: 30–87). Median follow-up period was 26 months (range: 3–99). Cavity formation was observed in 38 infarct lesions (61%). Eighteen lesions (29%) were partially adjacent to WMHs and 7 (11%) were fused into WMHs. In a multiple logistic regression analysis, age [odds ratio per 5-year increase: 1.34; 95% confidence interval (CI): 1.03–1.80; *p* = 0.03] and baseline infarct volume (odds ratio per 1-ml increase: 4.7; 95% CI: 1.6–19.7; *p* = 0.003) were independent predictors of cavity formation. There was a significant volume reduction between baseline and follow-up infarct lesions (median volume reduction rate: 44%).

**Conclusion:**

More than one-third of recent small subcortical infarcts do not lead to cavity formation and 40% of infarct lesions overlap with WMHs. Our data indicate the continuity between recent small subcortical infarcts and WMHs.

## Introduction

Lacunes of presumed vascular origin are distinguished from white matter hyperintensities (WMHs) of presumed vascular origin, and considered as an independent predictor of future stroke (1, 2) and cognitive impairment (3, 4). Lacunes are defined as small round or oval subcortical lesions with cerebrospinal fluid (CSF) isointense cavity on MRI (5–7), and often regarded as the consequence of previous recent small subcortical infarcts, so-called lacunar infarcts. However, knowledge concerning the course of morphological changes of this stroke subtype is limited. The incidence of cavity formation after infarct varies widely among studies, ranging from 28 to 94% (8–11). This discrepancy could be due to heterogeneous study design (e.g., combined use of CT and MRI), insufficient follow-up periods, and imprecise classification of this stroke subtype.

In this study, we examined the long-term evolution of recent small subcortical infarcts by using MRI and identified the incidence of cavity formation, the infarct volume and diameter reduction, and the positional relationship between infarct lesions and WMHs. We used the ASCO classification (12) in order to select the patients with definite recent small subcortical infarct. The terminology of the standards for reporting vascular changes on neuroimaging (STRIVE) (7) was used to describe the features of small vessel disease.

## Materials and Methods

### Standard protocol approvals, registrations, and patient consents

The local ethics committee approved the study.

### Subjects

**Figure 1:**
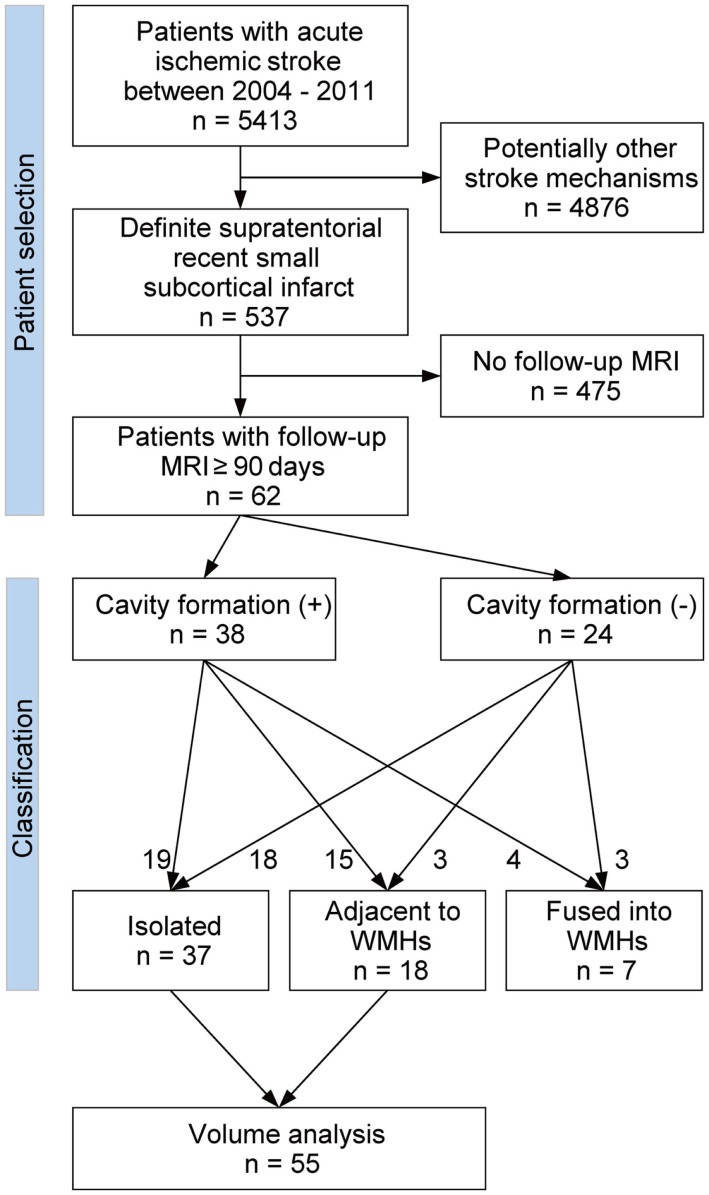
**Flow diagram of patient selection and classification**.

Figure 1 shows the flow diagram of patient selection and classification. From our prospectively collected stroke database, we identified 5413 acute symptomatic ischemic stroke patients who were admitted to our stroke unit between January 2004 and December 2011. Among these, 984 patients were clinically diagnosed with definite or possible supratentorial recent small subcortical infarct. We defined supratentorial recent small subcortical infarct as a single, round or oval, supratentorial subcortical ischemic lesion of increased signal on diffusion-weighted image (DWI) ≤20 mm distributed in the territory of lenticulostriate, choroidal anterior, thalamic, or perforating medullary branch. We included only the patients with the presence of a highly likely ASCO phenotype S (for small vessel disease) graded 1 or 2 (12). In addition, we excluded all patients with potentially concomitant stroke mechanisms (ASCO phenotype A for atherothrombosis 1–2, C for cardioembolism 1–2, or O for other causes 1–2). ASCO classification and the MRI pattern analysis were performed by two expert neurologists (Martin Griebe, Kristina Szabo). Using this approach, we identified 537 patients with definite supratentorial recent small subcortical infarct as reported previously (13). Out of these 537 patients, we included all patients (*n* = 62) who underwent a follow-up brain MRI at 90 days or longer after the onset of stroke. These MRIs were performed either as a routine follow-up examination or because of mild non-specific symptoms such as headache and dizziness. If the patients underwent more than one follow-up brain MRI, the first and the last follow-up MRIs were used in the analyses. If patients experienced more than one small subcortical infarct, only the first infarct was included.

### MRI assessment

**Figure 2:**
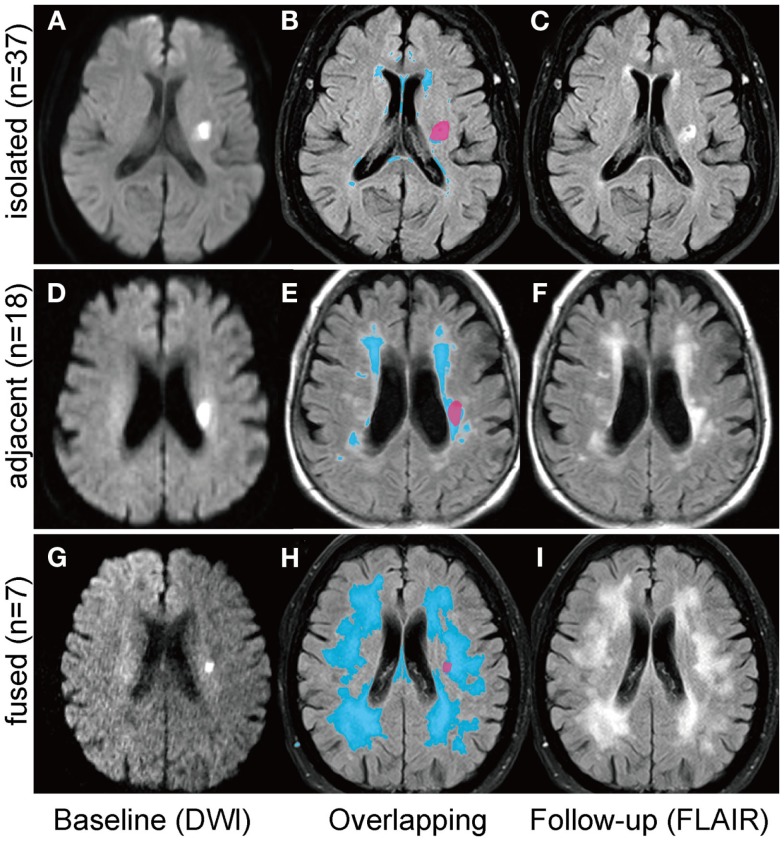
**Positional relationships between small subcortical infarcts and white matter hyperintensities**. Follow-up infarct lesions were categorized into three groups according to the positional relationships with white matter hyperintensities (WMHs); (1) “isolated”: the infarct lesion is isolated from WMHs **(A–C)**; (2) “adjacent”: less than 50% of the infract lesion overlaps with WMHs **(D–F)**; (3) “fused”: more than 50% of the infarct lesion overlaps with WMHs **(G–I)**. The left column shows the baseline diffusion-weighted images and the right column shows the follow-up FLAIR images. Overlapping images **(B,E,H)** are acquired by using 3D slicer software (http://www.slicer.org).

The brain MRI investigations were performed on 1.5- and 3.0-Tesla MRI scanners (MAGNETOM Sonata, Trio or Skyra, Siemens, Erlangen, Germany). The scanning protocol included transversal DWI, T2-weighted fluid-attenuated inversion recovery (FLAIR), T1- and T2-weighted images, and MR angiography. All axial scans were obtained with a 5-mm slice thickness. The baseline infarct volumes and diameters were measured on baseline DWI images; the follow-up infarct volumes and diameters were analyzed on the follow-up FLAIR images. A stroke-neurologist (Shuhei Okazaki), blinded to the clinical data, outlined borders of the lesions manually on each transverse DWI and FLAIR image, then calculated areas using the scanner software package (Syngo, Siemens, Erlangen, Germany). Areas of lesion on each slice were summed, and the sum was multiplied by the thickness of each slice for the volume. Additionally, the diameter of the infarct lesion on each transverse slice was measured and the largest diameter was collected. If the follow-up infarct lesions overlapped with preexisting WMHs, they were categorized as either “adjacent” (<50% of lesion volume overlaps with WMHs; Figures 2D–F) or “fused” (≥50% of lesion overlaps with WMHs; Figures 2G–I). Those patients with an infarct fused into WMHs were excluded from the volume analyses, because the border between infarct lesion volume and WMHs was unclear. The presence of cavity was defined as the appearance of a CSF isointense lesion on T1-weighted sequences within the original area of acute DWI abnormality. Two stroke neurologists (Shuhei Okazaki, Eva Hornberger) viewed T1-weighted sequences of follow-up MRI, and assessed the presence of a cavity independently blind to other MRI data. There was good agreement on the presence of cavity [κ = 0.84; 95% confidence interval (CI): 0.70–0.97]. Discrepancies were resolved by consensus. The volume of the cavity was measured similarly on the follow-up T1-weighted sequences. Baseline FLAIR sequences were further analyzed for WMHs using the Fazekas scale (14).

### Statistical analysis

Values are presented as median and interquartile range (IQR) for continuous and ordered variables, frequency and percentages for categorical variables. Baseline and follow-up infarct volumes and diameters were compared using paired *t*-test. Other values were compared using Mann-Whitney’s *U* test for continuous and ordered variables and Fisher’s exact test for categorical variables. Continuous and ordered variables of baseline characteristics were divided into tertiles and then the Cochran–Armitage test for trend was used to assess trends in the incidence of cavity formation. We performed a multivariate analysis with logistic regression to determine independent predictors of cavity formation with age, hypertension, and baseline infarct volume. All analyses were performed using JMP Pro Version 10.0.2 (SAS Institute Inc., Cary, NC, USA).

## Results

Among 537 patients with definite supratentorial recent small subcortical infarct, 62 patients (12%) underwent one or more follow-up MRIs after ≥90 days of stroke onset. These 62 patients were enrolled in this study. No significant differences in age, sex, baseline National Institute of Health Stroke Scale (NIHSS) score, past history of stroke, and the prevalence of atherosclerotic risk factors (hypertension, diabetes mellitus, dyslipidemia, and smoking habit) were observed between patients with (*n* = 62) and without follow-up MRI (*n* = 475). Table 1 shows the baseline characteristics of the patients. Median age was 71 years (range: 30–87 years) and 52% were male. All baseline MRIs were performed within 6 days (median: 32 h; range: 3–130 h), and the last follow-up MRIs were obtained on median 26 months (range: 3–99 months) after the stroke onset. Median baseline infarct volume was 0.53 ml (IQR: 0.25–1.21 ml) and median baseline infarct diameter was 10.1 mm (IQR: 6.9–15.4 mm).

**Table 1:** **Characteristics of patients with and without cavity formation on follow-up MRI**.

	Entire group	Cavity formation		
		Yes	No	
	*n* = 62	*n* = 38	*n* = 24	*p*-value
Age, years	71 [61, 77]	73 [68, 80]	66 [50, 71]	0.003*
Male	32 (52%)	20 (53%)	12 (50%)	1.00
Hypertension	54 (87%)	36 (95%)	18 (75%)	0.047*
Diabetes	18 (29%)	11 (29%)	7 (29%)	1.00
Dyslipidemia	33 (53%)	19 (50%)	14 (58%)	0.61
Smoking	16 (26%)	10 (26%)	6 (25%)	1.00
Past history of stroke	13 (21%)	10 (26%)	3 (13%)	0.34
Intravenous thrombolysis	8 (13%)	6 (16%)	2 (8%)	0.47
Infarct location
Basal ganglia or internal capsule	43(69%)	30 (79%)	13 (54%)	0.11
Subcortical white matter	6 (10%)	3 (8%)	3 (13%)	
Thalamus	13 (21%)	5 (13%)	8 (33%)	
Baseline Fazekas score	2 [1, 3]	2 [1, 3]	1 [0, 2]	0.06
Baseline lesion volume, ml	0.53 [0.25, 1.21]	0.80 [0.35, 1.35]	0.28 [0.09, 0.53]	0.001*
Baseline lesion diameter, mm	10.1 [6.9, 15.4]	11.7 [9.1, 16.0]	7.1 [6.3, 12.1]	0.003*
NIHSS admission	3 [2, 4]	4 [2, 5]	2 [1, 3]	<0.001*
Follow-up period, months	26 [12, 43]	26 [11, 48]	27 [13, 42]	0.76

Values are median (25th ‰, 75th ‰) or count (proportion). *p*-values were derived from univariate analyses. NIHSS indicates National Institute of Health Stroke Scale; *p < 0.05.

**Figure 3:**
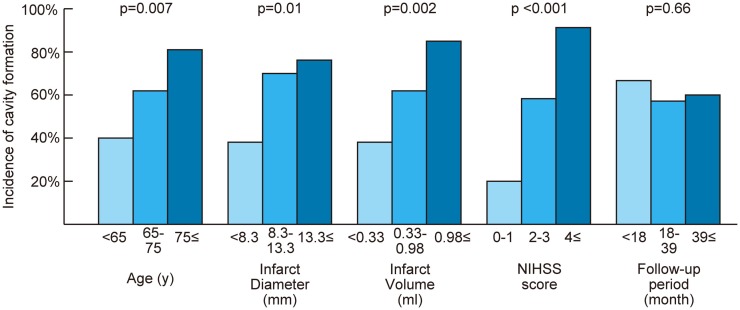
**Relationship between cavity formation and baseline characteristics**. Continuous values were divided into tertiles. The Cochran–Armitage test was performed to assess trends in the incidence of cavity formation. NIHSS indicates National Institute of Health Stroke Scale; *p* = *p* for trend.

Table 1 shows the characteristics of patients with and without cavity formation on follow-up MRI. Cavity formation, or formation of lacune of presumed vascular origin, was observed in 38 patients (61%). If a cavity was present, median volume of the cavity was 0.11 ml (IQR: 0.04–0.25 ml). One acute infarct lesion in the thalamus disappeared on the follow-up MRI after 8 months of stroke onset (tissue characteristics of thalamic and non-thalamic infarct were shown in Supplemantal Table 1). In univariate analyses, age, hypertension, baseline infarct volume, baseline infarct diameter, and baseline NIHSS score were significantly associated with the incidence of cavity formation (Table 1). Baseline NIHSS was closely related to baseline infarct volume (*r* = 0.44, *p* < 0.001) and baseline infarct diameter (*r* = 0.54, *p* < 0.001). In addition, continuous values were divided into tertiles and trends in the incidence of cavity formation were assessed (Figure 3). Cavities, or lacunes of presumed vascular origin, developed more often in elderly patients and in larger infarct lesions. In a multiple logistic regression analysis with age, hypertension, and baseline infarct volume; age (odds ratio per 5-year increase: 1.34; 95% CI: 1.03–1.80, *p* = 0.03) and baseline infarct volume (odds ratio per 1-ml increase: 4.7; 95%CI: 1.6–19.7, *p* = 0.003) were independent predictors of cavity formation. If a cavity was present, the cavity volume was closely related to baseline infarct volume (*r* = 0.71, *p* < 0.001), but not to age (*r* = −0.09, *p* = 0.58), time to follow-up (*r* = −0.10, *p* = 0.53), or preexisting risk factors such as hypertension, diabetes mellitus, dyslipidemia, smoking, and past history of stroke.

**Figure 4:**
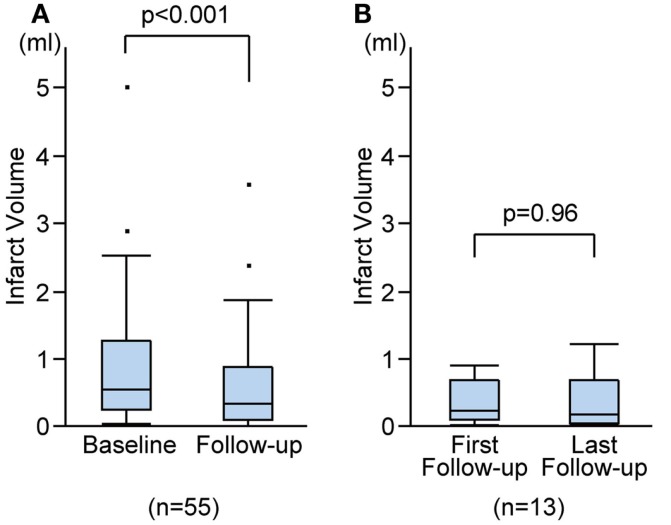
**Infarct volume changes during follow-up**. Infarct volume changes between baseline and the last follow-up MRIs **(A)**, and between the first and the last follow-up MRIs **(B)**.

On the follow-up MRI, 18 infarct lesions (29%) were located partially adjacent to preexisting WMHs and 7 (11%) were fused into WMHs (see Figures 1 and 2). Cavity formation was observed in 19 of 37 (51%) of isolated infarct lesions, 15 of 18 (83%) of lesions adjacent to WMHs, and 4 of 7 (57%) of lesions fused into WMHs. We excluded these seven patients with infarct lesions fused to WMHs from the volume analyses, because the border between infarct lesion and WMHs was unclear. Consequently, 55 patients were used for the volume analyses. The median follow-up infarct volume was 0.34 ml (IQR: 0.10–0.75 ml) and thus significantly smaller than the baseline infarct volume (*p* < 0.001, Figure 4A). The median volume reduction was 0.16 ml (IQR: 0.03–0.58 ml) and the median volume reduction rate was 44% (IQR: 9–57%). The median follow-up infarct diameter was 7.7 mm (IQR: 5.4–11.7 mm) and thus significantly smaller than the baseline infarct diameter (*p* < 0.001). The median diameter reduction was 2.0 mm (IQR: 0.7–4.1 mm) and the median diameter reduction rate was 21% (IQR: 9–34%). The absolute infarct volume reduction was strongly associated with the baseline infarct volume (*r* = 0.67, *p* < 0.001) and moderately with the baseline NIHSS score (*r* = 0.28, *p* = 0.04). On the other hand, it was not associated with age (*r* = −0.14, *p* = 0.32), time to follow-up (*r* = 0.07, *p* = 0.63), or preexisting risk factors.

Out of 55 patients, 13 underwent more than one follow-up MRI. The first follow-up MRIs were performed on median 15 months (range: 5–68 months) after stroke onset and the median duration between the first and the last follow-up MRIs was 24 months (range: 7–74 months). There was no difference in cavity formation between the first and the last follow-up MRIs. There was no significant difference between the first and the last follow-up infarct volume (median 0.27 ml, IQR 0.14–0.72 ml vs. median 0.22 ml, IQR 0.08–0.71 ml; *p* = 0.96; Figure 4B), or between the first and the last follow-up diameters (median 8.0 mm, IQR 6.2–10.4 mm vs. median 8.0 mm, IQR 6.0–10.4 mm; *p* = 0.96). The median volume reduction between the first and the last follow-up was 0.01 ml (IQR: −0.07 to 0.14 ml) and the median diameter reduction was 0.2 mm (IQR: −0.3 to 0.4 mm).

## Discussion

The main finding of the present study was that more than one-third (39%) of recent small subcortical infarcts developed no cavity, or lacune of presumed vascular origin, and 40% of infarct lesions located adjacent or fused in preexisting WMHs. Age and baseline infarct volume were independent predictors of cavity formation. These results indicate that a considerable number of recent small subcortical infarcts, especially smaller infarcts or those in younger patients, could be overlooked or misdiagnosed as primary WMHs at later stages of the disease. Commonly, recent small subcortical infarcts, or lacunar infarcts, are considered to result from acute severe ischemia of a single perforating artery, whereas WMHs are supposed to evolve from chronic diffuse hypoperfusion (15–17). However, our results demonstrate that initially delimited infarct lesions become indistinguishable from WMHs after some time. A similar continuity between recent small subcortical infarcts and WMHs was also reported in a study of cerebral autosomal dominant arteriopathy with subcortical infarcts and leukoencephalopathy (CADASIL) (18). These data suggested that the mechanisms underlying small subcortical infarcts and WMLs might overlap and that their pathogenesis is more closely connected.

Four previous studies evaluated the incidence of cavity formation after recent small subcortical infarcts. Koch et al. reported a similar incidence of cavity formation (23 of 38: 61%) by using MRI and CT (9), although they included larger infarcts (<25 mm) and did not exclude other stroke subtypes such as striatocapsular infarcts due to middle cerebral artery stenosis or occlusion of multiple perforating arteries by proximal embolism (19). Potter et al. reported that only 25 of 90 acute infarct lesions (28%) developed a cavity (8). However, the minimum follow-up period in that study was very short (6 days). Because the earliest time of definite cavity formation was 54 days after onset in their study, their follow-up period might have been insufficient to detect the eventual incidence of cavity formation. Different from these studies, Moreau et al. reported that 30 of 32 (94%) infarct lesions showed the evidence of cavity formation on T1 sequence at 90 days (10). However, the inter-rater agreement for the presence of cavity on T1 sequence was relatively low in this study (κ = 0.58, 95%CI: 0.33–0.83), and the incidence of definite cavity formation was 50–78% at 90 days. Loos et al. also reported a high incidence of cavity formation (77 of 82: 94%) (11); however, 41% of participants in their study had no evidence of acute DWI lesion on the baseline MRI. Because incidental asymptomatic lacunes are often identified in the brain imaging of acute stroke patients (20), there is a possibility that asymptomatic lacunes were misclassified as recent small subcortical infarcts, resulting in overestimation of the incidence of cavity formation. Thus, our rate of 61% fits well with all references mentioned, provided that similar time courses and definition for this stroke subtype were used.

Our results also indicate a significant volume and diameter reduction between the baseline and follow-up infarct lesions. Median volume and diameter reduction rate were 44 and 21%, respectively. Interestingly, our data show no associations between the duration of follow-up period (≥90 days) and the infarct volume reduction, the incidence of cavity formation, or the cavity volume. We also found no volume or diameter change between the first and the last follow-up infarct lesions. Moreau et al. reported that definite cavity formation was more common at 90 days than at 30 days (10). These findings suggest that a majority of morphological changes in small subcortical infarct occurs within the first 90 days.

Although this study had a relatively small sample size, the major strengths of our study are that all baseline and follow-up assessments were evaluated by using a standardized MRI-protocol, that recent small subcortical infarcts were strictly identified according to the phenomenology- and evidence-based ASCO classification (12), and that the follow-up period was sufficiently long to evaluate the development of cavity. However, our study also has limitations. First, follow-up MRIs were not performed per predefined protocol, but based on the treating physicians’ decision. Consequently, although there were no significant differences in baseline characteristics between patients with and without follow-up MRI, a selection bias may have been introduced. Second, we could not standardize the field strength (1.5T and 3.0T) of MRI due to the renewal of MRI scanners. As the higher field MRI might detect cavities more sensitively (10), the true incidence of cavity formation might be higher than our results. Finally, we measured the baseline infarct volume on DWI and follow-up infarct volume on FLAIR, because baseline FLAIR often showed no or only faint acute ischemic lesions. It has been reported that subacute FLIAR lesions are larger than acute DWI lesions mainly due to the progression of infarct and vasogenic edema (21, 22). Therefore, our results may underestimate the volume reduction of small subcortical infarcts.

In conclusion, we show that more than one-third of recent small subcortical infarcts do not lead to cavity formation and that 40% of infarct lesions overlap with WMHs. The incidence of cavity formation depends on the patient’s age and baseline infarct volume. These data indicate that initially delimited small subcortical infarct lesions become indistinguishable from WMHs. Further prospective research by combining neuroimaging with a pathological approach may elucidate the complex mechanisms underlying small subcortical infarcts, lacunes, and WMHs.

## Author Contributions

Study concept and design: MG, AG, MH, and KS. Acquisition, analysis, or interpretation of data: SO, EH, MG, and KS. Drafting of the manuscript: SO and EH. Critical revision of the manuscript for important intellectual content: MG, AG, MH, and KS.

## Conflict of Interest Statement

The authors declare that the research was conducted in the absence of any commercial or financial relationships that could be construed as a potential conflict of interest.

## Supplementary Material

The Supplementary Material for this article can be found online at http://journal.frontiersin.org/article/10.3389/fneur.2015.00118/abstract
