# Supplementary material for: MRI Characteristics of the Evolution of Supratentorial Recent Small Subcortical Infarcts
Source: Front Neurol. 2015 May 26;6:118. doi: 10.3389/fneur.2015.00118 (PMC4443727; doi:10.3389/fneur.2015.00118)
Supplement: Supplementary file 1 [file Table_1.DOCX]

|  | Thalamic | Non-thalamic | p-value |
| --- | --- | --- | --- |
| Number of patients | 13 | 49 |  |
| Baseline lesion volume, ml | 0.22 [0.09, 0.52] | 0.69 [0.30, 1.33] | 0.02 |
| Follow-up lesion volume, ml | 0.10 [0.05, 0.36] | 0.53 [0.15, 0.93] | <0.01 |
| Volume reduction, ml | 0.13 [0.02, 0.38] | 0.13 [0.00, 0.61] | 0.93 |
| Volume reduction rate, % | 56% [16%, 75%] | 38%, [0%, 57%] | 0.13 |
| Cavity formation | 5 (38%) | 33 (67%) | 0.11 |

**Supplemental Table 1. Tissue characteristics of thalamic and non-thalamic infarct.**

Values are median [25th percentile, 75th percentile] or count (proportion).
